# Genetic Variability of the AcrAB-TolC Multidrug Efflux Pump Underlies SkQ1 Resistance in Gram-Negative Bacteria

**DOI:** 10.32607/20758251-2019-11-4-93-98

**Published:** 2019

**Authors:** P. A. Nazarov, E. A. Kotova, V. P. Skulachev, Y. N. Antonenko

**Affiliations:** Belozersky Institute of Physico-Chemical Biology, Lomonosov Moscow State University, Moscow, 119991 Russia.; Mitotech LLC, Moscow, 119991 Russia; Institute of Mitoengineering, Lomonosov Moscow State University, Moscow, 119991 Russia

**Keywords:** SkQ1, AcrZ, AcrAB-TolC efflux pump, multidrug resistance

## Abstract

SkQ1, a novel antibiotic targeting bacterial bioenergetics, is highly effective against both gram-positive and gram-negative bacteria. However, some gram-negative bacteria, such as *Escherichia coli *and *Klebsiella pneumoniae*, are highly resistant to it. In different gram-negative bacteria, this resistance is associated with the identity of their AcrB transporter protein sequence with the sequence of the AcrB protein from *E. coli*. SkQ1 is expelled from *E. coli *cells by the AcrAB-TolC multidrug efflux pump. In this study, we demonstrate that SkQ1 resistance in *E. coli*, in contrast to chloramphenicol resistance, does not depend on the presence of the multidrug efflux pump accessory protein AcrZ.

## INTRODUCTION

**Fig. 1:**
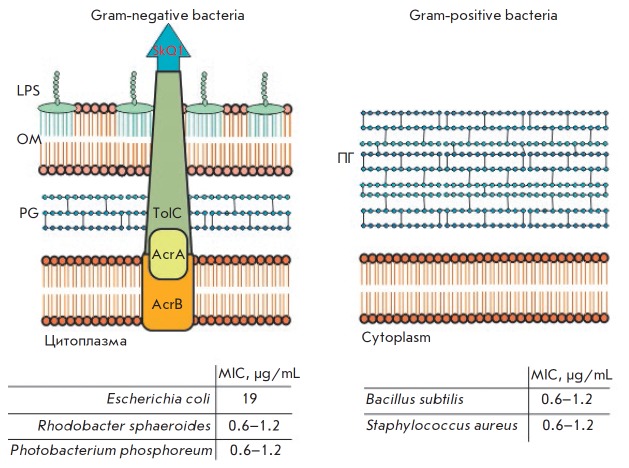
Schematic of the bacterial cell wall (LPS – lipopolysaccharides, OM – outer membrane, PG – peptidoglycan layer) and the antibacterial effect of SkQ1 against gram-positive and gram-negative bacteria. The sensitivity of gram-negative bacteria to SkQ1 depends on the structure of the protein components of the AcrAB-TolC pump

SkQ1, decyl triphenylphosphonium-conjugated plastoquinone, is a member of a new class of antibiotics that directly affect bacterial bioenergetics. The SkQ1 ability to inhibit growth of a variety of gram-negative and gram-positive bacteria may be used in medicine and agriculture; therefore, it is important to study its effect on microbial ecosystems and the development of resistance to it. We have demonstrated [1, 2] that SkQ1 resistance in *E. coli* is due to the presence of a specific multidrug resistance (MDR) pump AcrAB-TolC *(Fig. 1)* that underlies resistance to a wide range of antibiotics, surfactants, bile salts, pigments, and small organic molecules [3]. However, our study [1] did not analyze all TolC-dependent pumps, namely the putative TolC-dependent pump EmrKY-TolC, EntS, and the protein AcrZ. The small accessory protein AcrZ (also known as YbhT) of 49 amino acid residues is known to bind to the AcrAB-TolC complex, which comprises the AcrA, AcrB, and TolC proteins, and enhance the pump ability to remove certain classes of substrates from the cell: e.g., tetracycline, puromycin, and chloramphenicol [4].

Bacteria have genetic plasticity, which allows them to respond to a wide range of environmental threats, such as antibiotics. Bacteria use two main genetic survival strategies: (1) acquisition of resistance determinants through horizontal gene transfer and (2) mutations associated with antibiotic targets [5]. The amino acid sequences of the AcrA, AcrB, and TolC proteins are identical in laboratory *E. coli *B and K-12 sub-strains [6]. Previously, we demonstrated that removal of any of the AcrA, AcrB, or TolC proteins led to a complete loss of SkQ1 resistance [1]. The distance between the TolC and AcrB operons in the *E. coli *chromosome is about 175 kbp [7]; therefore, the likelihood of acquiring AcrAB-TolC pump-mediated resistance through interspecific horizontal gene transfer is very negligible.

To date, MDR pump-mediated resistance is the only known mechanism of SkQ1 resistance, and AcrAB-TolC is the only known pump that removes SkQ1 from the cell. Based on the data on the ability of the small protein AcrZ to regulate resistance to antibiotics, such as tetracycline, puromycin, and chloramphenicol [4], it may be supposed that SkQ1 resistance is also modulated by AcrZ. On the other hand, SkQ1 resistance might be modulated by local and global transcriptional regulators, as well as through post-transcriptional and post-translational regulation [8].

## EXPERIMENTAL

The standard laboratory *E. coli *strains MG1655 and W3110 (F-lambda-IN (rrnD-rrE) 1 rph-1) were used in the study. The *E. coli *strains MC1061, DH5α, and BL21 (DE3) were provided by S.S. Sokolov (Belozersky Institute of Physico-Chemical Biology, Moscow State University); the *E. coli *strain JM109 was provided by L.A. Novikova (Belozersky Institute of Physico-Chemical Biology, Moscow State University); the *E. coli *strain GR70N was received from Yu.V. Bertsova (Belozersky Institute of Physico-Chemical Biology, Moscow State University); and the *E. coli *strain XL1-Blue was purchased from Eurogen company (Moscow, Russia).

The *E. coli *deletion strains ECK0751 (devoid of the *acrZ *gene), ECK0584 (devoid of the *entS *gene), ECK2363 (devoid of the *emrY *gene), ECK2364 (devoid of the *emrK *gene) were kindly provided by H. Niki (National Institute of Genetics, Japan) [9].

*Staphylococcus aureus *was received from the microorganisms collection of Lomonosov Moscow State University (No. 144). *Photobacterium phosphoreum *was provided by A.D. Ismailov (Belozersky Institute of Physico-Chemical Biology, Moscow State University). *Rhodobacter sphaeroides *was provided by G. Klug (Institute for Microbiology and Molecular Biology at Justus-Liebig-University of Giessen, Germany).

Bacterial cells were grown at 37°C in LB or a Mueller–Hinton medium at a shaking rate of 140 rpm as described in [1].

SkQ1 resistance was studied by double dilutions in a liquid nutrient medium using home-made panels according to the Clinical and Laboratory Standards Institute (CLSI) recommendations. Mueller–Hinton broth (HIMEDIA, Mumbai, India) was used in the study. A dilutions panel was prepared in a 96-well microtiter plate in a volume of 200 μL per well. A bacterial suspension (50 μL) in Mueller–Hinton broth was added to each well to a final suspension volume of 250 μL (5 × 10^5^ CFU/mL). The resulting suspension was incubated at 37°C for 20 h [1].

The minimum inhibitory concentration (MIC) was determined as the lowest concentration completely inhibiting bacterial growth. Bacterial growth was observed visually, along with OD_620_ measurements [1].

For bioinformatics analysis, we used the BLASTp search tool (NCBI, https://blast.ncbi.nlm.nih.gov), STRING v.10.5 database (EMBL, http://string.embl. de/), and BioCyc database from the Pathway/Genome Database Collection (PGDBs, https://biocyc.org/).

## RESULTS AND DISCUSSION

We compared the resistance of various *E. coli *laboratory strains and found that all these strains were resistant to SkQ1 (*Table 1*). This is apparently explained by the identity of the primary structure of the AcrA, AcrB, and TolC proteins in all the studied strains [6].

**Table 1:** Bacterial susceptibility to SkQ1: measurements of the minimum inhibitory concentration (MIC). Comparison of SkQ1 activity against Staphylococcus aureus with that of various antibiotics under identical conditions

Bacterium	Antibiotic	MIC, µg/mL	Reference
E. coli strain			
W3110	SkQ1	19	[1]
MG1655	SkQ1	19	Present study
JM109	SkQ1	19	«
BL21(DE3)	SkQ1	19	«
XL1-Blue	SkQ1	19	«
DH5α	SkQ1	19	«
MC1061	SkQ1	19	«
GR70N	SkQ1	19	«
Deletion E. coli MG1655 strains			
AcrD, AcrE, AcrF, MacA, MacB, MdtA, MdtB, MdtC, MdtE, MdtF, EmrA, EmrB	SkQ1	19	[1]
AcrZ, EmrK, EmrY,EntS	SkQ1	19	Present study
AcrA, AcrB, TolC	SkQ1	0.6–1.2	[1]
R. sphaeroides	SkQ1	0.6–1.2	Present study
P. phosphoreum	SkQ1	0.6–1.2	«
K. pneumoniae	SkQ1	>19	«
S. aureus	SkQ1	0.6–1.2	Present study, [1]
	Kanamycin	2.5	Present study
	«	3.1	[10]
	Chloramphenicol	5	Present study
	«	3.1	[10]
	Ampicillin	2.5	Present study
	«	1.6	[10]
	Streptomycin	6.3	[10]
	Polymyxin B	100	[10]

Earlier [1], we showed that the gram-negative bacteria *P. phosphoreum *and *R. sphaeroides*, unlike *E. coli *strains, were not resistant to SkQ1. According to the data given in *Table 2*, the amino acid sequence of the proteins, annotated as AcrB, from these bacteria is quite different from the sequence of the AcrB protein from *E. coli*. The levels of their identity with the *E. coli *AcrB protein are 65 and 33%, respectively, which apparently manifests itself in a rather high sensitivity of these bacteria to SkQ1. Of note, the AcrD protein, removal of which does not affect SkQ1 sensitivity, is 66% identical to the AcrB protein sequence, which is comparable to the AcrB proteins from *P. phosphoreum *and *R. sphaeroides*. Therefore, SkQ1 resistance in bacteria requires a higher similarity of the amino acid sequence of their AcrB protein to the *E. coli *AcrB protein sequence. To examine this conclusion, we determined the primary structure of the AcrB protein from another gram-negative bacterium, *Klebsiella pneumoniae*, which was found to be 91.5% identical to the *E. coli *AcrB protein structure. This suggested the presence of SkQ1 resistance in *K. pneumoniae*, which was confirmed experimentally (*Tables 1 *and *2*).

**Table 2:** Comparison of the acrB gene sequences from different strains of gram-negative bacteria with the acrB sequence from the E. coli strain

Bacterium	Identification number	Overlap, %	Identity, %	Resistance to SkQ1
E. coli MG1655	NP_414995.1	100	100	YES
E. coli W3110	BAE76241.1	100	100	YES
E. coli AcrB*	NP_416965.1*	99	66	NO
E. coli BL21(DE3)	CAQ30935.1	100	100	YES
E. coli DH5α	KGA88788.1	100	100	YES
R. sphaeroides	ANS33442.1	97	33	NO
P. phosphoreum	CEO37741.1	98	65	NO
K. pneumoniae	CDO13174.1	99	91.5	YES

Note. In the case of the E. coli AcrB deletion mutant MG1655, denoted by an asterisk, comparison was performed with the AcrD protein sequence. The amino acid sequence identity was defined as the percentage of identical amino acid residues at the corresponding positions in aligned sequences. Overlap was defined as the percentage of aligned AcrB protein sequences. The absence (NO) and presence (YES) of resistance to SkQ1 was determined with respect to E. coli, where MIC of SkQ1 comparable to MIC of SkQ1 for E. coli was a criterion for resistance.

**Fig. 2:**
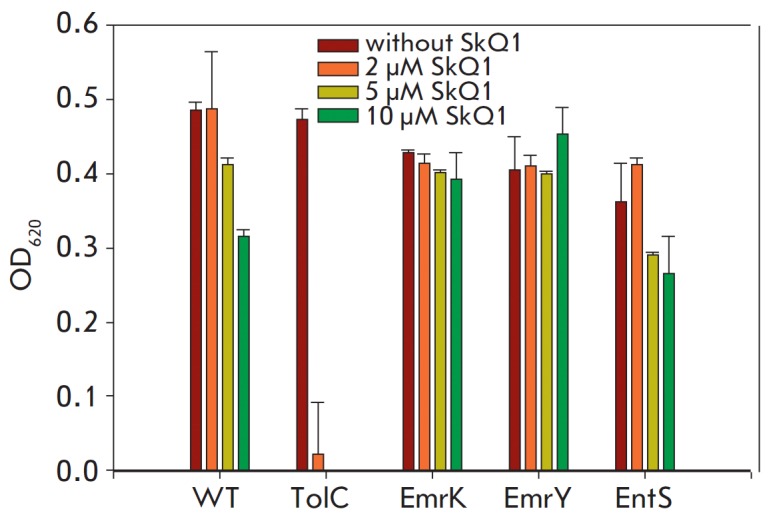
Toxicity of SkQ1 against the *E. coli *MG1655 strain and its deletion mutants ΔTolC (positive control), ΔEmrK, ΔEmrY, and ΔEntS. SkQ1 (2–10 μM) was added to bacterial cultures (1–5 × 10^5^ cells/mL) placed in 96-well plates. Cell density was determined by absorption at 620 nm. After that, bacteria were allowed to grow at 37°C for 20 h and the cell density was again measured. Data are presented as a mean value ± standard deviation for at least three experiments

An analysis of the SkQ1 antibacterial activity in *E. coli *mutants with deletions of the EmrK, EmrY, and EntS proteins (*Fig. 2*) revealed that the minimum inhibitory concentrations of SkQ1 were the same as those determined for the wild-type *E. coli* strain (*Table 1*).

To elucidate the role of the AcrZ protein in *E. coli *resistance to SkQ1, we compared the resistance of the wild-type *E. coli *MG1655 strain and that of strains with deletions of the AcrZ and AcrB proteins. If the AcrZ protein is involved in the AcrAB-TolC MDR pump functioning with formation of the AcrABZ–TolC complex, then removal of SkQ1 requires that the stability of the AcrZ protein deletion mutant be higher than that of the AcrB protein deletion mutant but lower than that of the wild-type protein. If the AcrZ protein is not involved in the AcrAB-TolC pump functioning, then the resistance of the AcrZ protein deletion mutant should be the in the wild-type strain and higher than in the case of an AcrB protein deletion. As a positive control in these experiments, we used chloramphenicol [10], removal of which from the cell is enhanced by the AcrZ protein [4]. The AcrZ protein impact on resistance to SkQ1 and chloramphenicol was determined simultaneously to exclude the impact of experimental conditions on the obtained result.

**Fig. 3:**
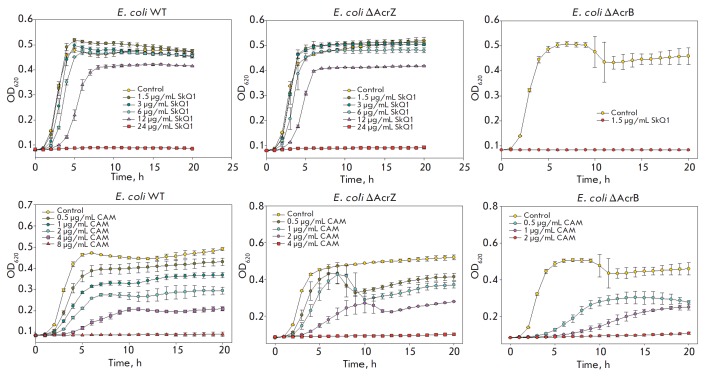
Effect of SkQ1 (upper panel) and chloramphenicol (CAM) (lower panel) on growth of *E. coli *bacteria (WT, ΔAcrB, and ΔAcrZ). SkQ1 (2.5–40 μM) or chloramphenicol (0.5–8 μg/mL) was added to the bacterial cultures (5 × 10^5^ cells/mL) placed in 96-well plates. Growth was assessed by hourly measured absorbance at 620 nm on a Multiskan FC plate reader (Thermo Fisher Scientific) during incubation. Bacteria were incubated at 37°C for 20 h. Data points are mean value ± standard deviations for at least three experiments

In our experiments, the AcrZ protein deletion mutant exhibited SkQ1 resistance similar to that in wild-type *E. coli *strains (*Fig. 3*), while three *E. coli *strains (WT, ΔAcrZ, and ΔAcrB) demonstrated different levels of resistance to chloramphenicol (*Fig. 3*), as described previously [4]. According to [4], binding of AcrZ to AcrB may cause conformational changes in its periplasmic domain, which affects recognition and capture of low hydrophobic substrates. Because SkQ1 is a highly hydrophobic compound (logP = 4.11) [11, 12], its recognition by the pump may not be regulated by the binding of AcrZ to AcrB.

An analysis of deletion mutants revealed that the EmrKY-TolC pump is not involved in the expelling of SkQ1 from the bacterial cell. Removal of the *entS *gene also had no effect on the expelling of SkQ1 from the bacterial cell. Thus, our conclusion that AcrAB-TolC was the only pump expelling SkQ1 was confirmed.

Another possible modulator of resistance to SkQ1 may be 6S RNA, a regulator of sigma-70-dependent gene transcription [13]. Our preliminary studies did not reveal differences in SkQ1 resistance between an *E. coli *SsrS protein deletion mutant and the wild-type *E. coli *strain. It cannot be ruled out that resistance to antibiotics targeting bacterial bioenergetics, such as SkQ1, may be enhanced by a trivial increase in the expression level. Expression of pleiotropic drug resistance pumps in *Saccharomyses cerevisiae *yeast was recently shown [13] to be induced by dodecyl triphenylphosphonium, another member of this class of antibiotics: i.e., dodecyl triphenylphosphonium can act as both an activator and an inhibitor of drug resistance [14, 15]. However, there is no direct correlation between temporal activation of expression and a constant increase in pleiotropic resistance to these compounds.

## CONCLUSION

Therefore, these findings indicate that SkQ1 is an effective antibiotic; SkQ1 resistance in *E. coli *bacteria is associated only with the AcrAB-TolC pump. An essential factor underlying SkQ1 resistance in other gram-negative bacteria is the identity of their AcrB proteins to AcrB from *E. coli*. The AcrZ protein is not involved in the development of SkQ1 resistance; in other words, the routine way to regulate resistance by affecting the AcrAB-TolC MDR pump through the AcrZ protein is ineffective in the case of SkQ1.

## References

[1] Nazarov P.A., Osterman I.A., Tokarchuk A.V., Karakozova M.V., Korshunova G.A., Lyamzaev K.G., Skulachev M.V., Kotova E.A., Skulachev V.P., Antonenko Y.N. (2017). Sci. Re.

[2] Khailova L.S., Nazarov P.A., Sumbatyan N.V., Korshunova G.A., Rokitskaya T.I., Dedukhova V.I., Antonenko Y.N., Skulachev V.P. (2015). Biochemistry (Mosc.)..

[3] Pos K.M. (2009). Biochim. Biophys. Acta..

[4] Hobbs E.C., Yin X., Paul B.J., Astarita J.L., Storz G. (2012). Proc. Natl. Acad. Sci. USA..

[5] Munita J.M., Arias C.A. (2016). Microbiol. Spectr..

[6] Karakozova M.V., Nazarov P.A. (2018). Bull. RSMU..

[7] Keseler I.M., Mackie A., Peralta-Gil M., Santos-Zavaleta A., Gama-Castro S., Bonavides-Martínez C., Fulcher C., Huerta A.M., Kothari A., Krummenacker M. (2013). Nucleic Acids Research.

[8] El Meouche I., Dunlop M.J. (2018). Science..

[9] Baba T., Ara T., Hasegawa M., Takai Y., Okumura Y., Baba M., Datsenko K.A., Tomita M., Wanner B.L., Mori H. (2006). Mol. Syst. Biol..

[10] Sabath L.D., Garner C., Wilcox C., Finland M. (1976). Antimicrob. Agents Chemother..

[11] Martyushin A.A., Tsarev D.A., Grigorenko M.A., Fedorov I.I., Ramenskaya G.V., Tashlitsky V.N., Skulachev V.P. (2008). Pharmacia..

[12] Antonenko Y.N., Avetisyan A.V., Bakeeva L.E., Chernyak B.V., Chertkov V.A., Domnina L.V., Ivanova O.Y., Izyumov D.S., Khailova L.S., Klishin S.S. (2008). Biochemistry (Mosc.)..

[13] Wassarman K.M. (2018). Microbiol. Spectr..

[14] Galkina K.V., Besedina E.G., Zinovkin R.A., Severin F., Knorre D. (2018). Sci. Rep..

[15] Knorre D.A., Markova O.V., Smirnova E.A., Karavaeva I.E., Sokolov S.S., Severin F.F. (2014). Biochem. Bio.

